# Optimizing TELSAM Fusion Constructs for Enhanced Protein Crystallization: Effects of Display Density and His Tag Configuration

**DOI:** 10.1063/4.0001132

**Published:** 2025-10-27

**Authors:** Prasadika Samarawickrama Hetti Arachchige, Kyle Ludlow, Dallin Mead, Alexis Xiong, James Moody

**Affiliations:** Brigham Young University, Provo, Utah, USA

## Abstract

With the numerous diseases and maladies that afflict mankind, there is a high demand for drugs that can combat their effects with great precision. Crystallography is a powerful tool that enables visualization of target proteins and their interactions with drug candidates at atomic resolution. However, a major limitation of protein crystallography is that most proteins do not readily crystallize independently; only about 30% are naturally crystallizable, limiting the utility of current visualization methods (Chen L, 2004).

The Human Translocation ETS Leukemia protein Sterile Alpha Motif (TELSAM) is a protein crystallization chaperone that has shown promise in aiding the crystallization of recalcitrant proteins. It is purposefully designed to be soluble at high pH and to polymerize at low pH, making it particularly useful for controlled crystallization (Kim CA, 2001; Poulos S, n.d.). Because both TELSAM and its usage conditions can be manipulated to maximize efficiency, it is a versatile tool in protein crystallography.

Previous studies have demonstrated that TELSAM can significantly enhance the crystallization propensity of target proteins compared to the proteins alone (Nawarathnage S, n.d.). In this study, we explored the effect of displaying 2, 3, or 6 copies of either a 9 kDa UBA domain or a 19 kDa vWA domain per turn of the TELSAM polymer. A flexible linker was used between TELSAM and the target proteins. In the 1TEL construction, there are 6 target proteins per turn; in 2TEL and 3TEL, there are 3 and 2, respectively.

To assess the effect of the 10x histidine (His) tag on crystallization, each construction was made with either a permanent or cleavable His tag. Protein crystals were successfully obtained for all UBA and vWA constructs. These crystals were analyzed and compared in terms of crystallization time, crystallization propensity, crystal size and shape, crystal lattice architecture, structure refinement statistics, diffraction resolution, fraction of reflections indexed, mosaicity, presence of twinning or broken periodicity, and ease of structure determination.

We found that 1TEL-UBA fusions crystallized within 24 hours and performed best without the His tag. In contrast, the 2TEL and 3TEL constructs crystallized better with the His tag. Among all constructs, the 1TEL fusions outperformed others in terms of crystal size and diffraction resolution. Notably, the 1TEL-vWA construct achieved the highest resolution (1.2 Å) observed for this protein and TELSAM.

We deposited the structures of His-1TEL-vWA (PDB: 9DOC), His-2TEL-UBA (PDB: 9CPL), and 3TEL-UBA (PDB: 9O0H) in the Protein Data Bank. These structures allowed for a detailed comparison of the crystal architecture among different TELSAM fusions. The presence of a His tag was found to have either a positive or negative effect, depending on the display density and the specific target protein.

This study enables us to recommend the most suitable TELSAM fusion strategy for crystallizing proteins of interest. These insights enhance the utility of TELSAM fusion crystallography, accelerating structural biology research and paving the way for novel approaches in drug design.

